# Role of Extracellular Vesicles in Influenza Virus Infection

**DOI:** 10.3389/fcimb.2020.00366

**Published:** 2020-07-21

**Authors:** Yuan Jiang, Xiaowen Cai, Jiwen Yao, Huanhuan Guo, Liangjun Yin, Wingnang Leung, Chuanshan Xu

**Affiliations:** ^1^Key Laboratory of Molecular Target and Clinical Pharmacology, State Key Laboratory of Respiratory Disease, School of Pharmaceutical Sciences and Fifth Affiliated Hospital, Guangzhou Medical University, Guangzhou, China; ^2^Department of Rehabilitation Medicine, The First Affiliated Hospital of Chengdu Medical College, Chengdu, China; ^3^Department of Orthopedic Surgery, The Second Affiliated Hospital, Chongqing Medical University, Chongqing, China; ^4^Asia-Pacific Institute of Aging Studies, Lingnan University, Tuen Mun, China

**Keywords:** influenza virus, extracellular vesicles, exosomes, immune response, vaccine

## Abstract

Influenza virus infection is a major health care concern associated with significant morbidity and mortality worldwide, and cause annual seasonal epidemics and pandemics at irregular intervals. Recent research has highlighted that viral components can be found on the extracellular vesicles (EVs) released from infected cells, implying a functional relevance of EVs with influenza virus dissemination. Therefore, exploring the role of EVs in influenza virus infection has been attracting significant attention. In this review, we will briefly introduce the biogenesis of EVs, and focus on the role of EVs in influenza virus infection, and then discuss the EVs-based influenza vaccines and the limitations of EVs studies, to further enrich and boost the development of preventative and therapeutic strategies to combat influenza virus.

## Introduction

Influenza virus, a single-stranded negative-sense RNA virus, belongs to *Orthomyxoviridae* family, and can infect the nasal and tracheal airways, and then spread throughout the upper and lower respiratory tract (Tumpey and Belser, 2009). Influenza virus infection usually causes various syndromes such as fever, cough, headache, body ache, runny nose and even severe pneumonia depending largely on the health status and immunologic function of the patient and virus pathogenicity. Seasonal influenza and pandemic influenza are manifestations of influenza virus infection in human beings, moreover, avian-origin influenza virus occasionally jumped species and spread to human successfully, leading to limited, non-sustained human-to-human avian influenza virus transmission, and the mortality is high (Malik et al., 2009). During the influenza season, according to statistics, 5–15% of the human population could be infected, causing 250,000–500,000 deaths a year worldwide (Ginsberg et al., 2009; Goeijenbier et al., 2014). Since 2010, 140,000–710,000 inpatients of the United States were infected with influenza viruses, and 12,000–56,000 deaths each year (McGowan et al., 2019). Most seasonal influenza virus infections are self-limiting, however, they can cause serious or fatal pulmonary dysfunction and even acute respiratory distress syndrome (ARDS) (Khatri et al., 2018). Meanwhile, the oversecretion of inflammatory cytokines (known as “cytokine storm”) induced by the immune or inflammatory response, aggravate the damage degree of ARDS, even induce many organs failures and finally raise the death risk (Tisoncik et al., 2012). Moreover, during or shortly after influenza virus infection, patients usually face increased risk of thrombosis-related cardiovascular events, such as myocardial infarction and stroke (Warren-Gash et al., 2009; Antoniak et al., 2016). Dreadfully, a new strain or variant of influenza virus might result in a pandemic with millions of fatalities.

According to the different antigenicity of the nucleoprotein (NP) and matrix protein (M), influenza viruses are usually classified into three different serotypes: influenza A virus (IAV), influenza B virus (IBV), and influenza C virus (ICV) (Zheng et al., 2020). IAV has genetically distinct subtypes based on 18 hemagglutinin (HA) and 11 neuraminidase (NA) surface glycoproteins. Some subtypes of IAV have established stable transmission profiles among birds, swine, and humans (Huo et al., 2019). In particular H5N1 and H7N9 are highly pathogenic subtypes of avian origin, and directly transmitted from birds to humans with highly contagious and widespread outbreak patterns (Li et al., 2015, 2017). H1N1 is the earliest emerging subtype with available genomic sequences, has caused several pandemics and seasonal epidemics, resulting in millions of deaths and enormous economic losses (Yin et al., 2018). Once influenza virus settle in the respiratory tract of humans, the virus can break the mucus barriers and rapidly infect the primary host cells (i.e., epithelial cells) to release the genome segments into the cell to start virus transcription and replication. First of all, influenza viruses bind to sialic acid receptor on the surface membrane of the epithelial cells using HA, and then enters into host cells via viropexis and/or receptor-mediated endocytosis process. Viropexis is the predominant metabolical activity-independent mechanism of influenza viruses attachment envelopment and the subsequent formation of virus-containing intracellular vacuoles for entry into the host cell (Patterson et al., 1979). The virus particles are encapsulated into endosomes, and transported to locations near the cell nucleus. After the viral membranes fused with endosomes, viral ribonucleoprotein complex (vRNPs) as the templates are released into the cell nucleus to synthesize messenger RNA (mRNA) and complementary RNA (cRNA) for viral translation and replication. Newly synthesized vRNPs are assembled and exported from the cell nucleus and then directed to the plasma membrane. Finally, vRNPs are incorporated into budding virions and released into extracellular environment (Keshavarz et al., 2018), and then spread to uninfected cells or local immune cells such as macrophages (MΦ), which are the principal effector cells of the innate immune system (Cypryk et al., 2016). The intercellular transfer of viral materials released from infected cells to neighboring and distant recipient cells affect virus spread and pathogenesis. Importantly, this kind of cell- cell communication can directly activate host response to influenza virus infection, and modulate the host homeostasis (Assil et al., 2015).

It was widely believed that cell-cell communication is critical for maintaining homeostasis in body under many different physiological and pathologic conditions, not just viral infection, and can be mediated by direct cell-cell contact via transferring some soluble factors including hormones, cytokines, and inflammatory mediators (Schorey and Harding, 2016). Extracellular vesicles (EVs), a heterogeneous group of natural membrane vesicle released from various cells, are found in the plasma and other body fluids, such as saliva, sputum, urine, semen, and breast milk. EVs are initially regarded as cellular waste (Kalamvoki and Deschamps, 2016; Pleet et al., 2018), however, recent research has highlighted that EVs can also as an important mediator play autocrine/paracrine role in intercellular communication (Fujita et al., 2018). EVs attach to the surface of recipient cells by means of adhesion molecules, and then release their contents (e.g., proteins, lipids, and RNAs) into cytosol of recipient cells by phagocytosis, endocytosis, and macropinocytosis or direct fusing with cell membrane (Villarroya-Beltri et al., 2014; Tkach and Théry, 2016). Currently, EVs are called by different names in many articles according to their size and biogenesis, but only three main classes of EVs have been indentified, namely exosomes, microvesicles/ectosomes and apoptotic bodies (Hessvik and Llorente, 2018; Vidal, 2019). Exosomes, <150 nm in diameter, are released into the extracellular environment after fusion of multivescular bodies (MVBs) with the plasma membrane. Nevertheless, microvesicles and apoptotic bodies, both larger than 100 nm in diameter, are directly formed by plasma membrane outward budding and then released from living and dying cells, respectively (Palmulli and van Niel, 2018).

Many *in vivo* infection studies have shown that an elevated concentration of blood EVs was along with infection, and viral components (e.g., proteins and genome) were found on the EVs released from infected cells, demonstrating a functional relevance of these vesicles with virus dissemination in the body during an infection (Schorey and Harding, 2016). Recently, the relationship of EVs and viral infection has also been extensively investigated, including human immunodeficiency virus (HIV), hepatitis B virus (HBV), Hepatitis C virus (HCV), human papilloma virus (HPV), Epstein-Barr virus (EBV), human T-lymphotropic virus (HTLV), and members of the herpesvirus family (Meckes, 2015; Cone et al., 2019). Studies confirmed that EVs encapsulating viral materials could stimulate host response in the absence of direct infection of cells. For example, HIV-1 accessory protein Nef targets the MAL, a tetraspanning membrane protein, and then exploits MAL-dependent passway of exosome biogenesis for hijacking exosome release into Jurkat T cells to educate quiescent T cells permissive to HIV-1 replication (Ventimiglia et al., 2015). Moreover, EVs released from virus infected cells could also interact with non-immune cells such as hepatocytes for persistent infection (Devhare et al., 2017). Based on the above, not surprisingly, viruses have evolved to hijack the biosynthetic machinery of EVs as an astute survival strategy. The sorting and release of virion or viral components have to use or require the endosomal-sorting complexes required for transport (ESCRT) pathway (Schorey and Harding, 2016), as well as exosome and other EVs, implying that there exist some similarities between viral assembly and biogenesis of EVs in host cells. The EVs carrying viral proteins and genetic material, play a significant role in viral infection (Nolte-'t et al., 2016).

In this review, we will briefly introduce the biogenesis of EVs, and focus on the role of EVs in influenza virus infection, and then discuss the EVs-based influenza vaccine and the limitations of EVs studies, to further enrich and boost the development of preventative and therapeutic strategies to combat influenza virus.

## Similarity in EVs Biogenesis and Release of Influenza Virus Particles

Exosomes are the smallest one of EVs with round double membrane structure in appearance (Hessvik and Llorente, 2018). As the best-characterized subclass of EVs, exosomes have been arouseing general concern and widely researched. Besides mature red blood cells, nearly all eukaryotic cells can secrete exosomes depending on their endocytic capacities (Vidal, 2019). Exosomes are the only EVs formed from internal membrane, and the biogenesis of them starts from early endosomes mature into late endosomes, and budding into intraluminal vesicles (ILVs) after encapsulating selected cargo composed of proteins, nucleic acids, and other bioactive molecules (Anderson et al., 2016). ILVs are a direct result from parts of the limiting membrane bud into the lumen of the endosome, and encapsulated into mulitivesicular bodies (MVBs) (Schöneberg et al., 2017). MVBs are fused with autophagosomes for delivering them to lysosomes, or directly fused with lysosomes where their contents can undergo degradation, or directly transported to fuse with the plasma membrane for ILVs release to extracellular environment as exosomes (Gruenberg, 2020; Kalluri and LeBleu, 2020). The above process can be regulated through ESCRT-dependent pathway or ESCRT-independent pathways (e.g., Rab GTPases, tetraspanin complexes and ceremide pathway; Tkach et al., 2018). The ESCRT machinery consists of approximately thirty proteins, and those proteins assemble into core four complexes (e.g., ESCRT-0, -I, -II, and -III) and some associated proteins (e.g., VPS4, ALIX, and VTA1), especially the ESCRT-II subunits can bind to RNA resulting in the cytoplasmic mRNA and miRNA to sort into exosomes during the biogenesis process (Kouwaki et al., 2017).

Microvesicles are also named as ectosomes or microparticles, and less known in their biogenesis process compared to exosomes. In general, microvesicles are formed by direct budding of the outward plasma membrane via ARF6 and RHOA-dependent rearrangement of the actin cytoskeleton (Li et al., 2012), and Ca^2+^-activated scramblases (Cocucci and Meldolesi, 2015). The formation of microvesicles seems to be affected by some other mechanisms in different cells. In cancer cells, Rab GTPases Rab5 locating at the plasma membrane of cells, might play vital roles in microvesicles biogenesis (Das et al., 2018). Rab22a and hypoxia-inducible factors (HIFs) can also regulate microvesicles formation in the cancer cells under hypoxia condition (Wang et al., 2014). The ESCRT-III Pathway participates in microvesicles formation in cardiomyocytes. Like exosomes, microvesicles can also transfer mRNA and miRNAs to recipient cells, however, the mechanisms underlying the sorting of RNAs in microvesicles biogenesis remain unclear.

Apoptotic bodies are the only EVs formed during programmed cell death (PCD) (Ståhl et al., 2019). Apoptotic bodies biogenesis starts from budding of the outward plasma membrane through apoptotic pathway. After the critical morphological changes of apoptotic cells (e.g., membrane blebbing and membrane protrusion), apoptotic bodies encapsulateing chromatin, low molecular weight RNA, glycosylated proteins, nuclear fragments and even intact mitochondria, and are ultimately released into extracellular environment as a product of apoptotic cell disassembly. Moreover, apoptotic bodies not only modulate the response of immune system, but also transfer bioactive molecules to recipient cells that represent certain signaling pathway in cell-cell communication (Hristov et al., 2004).

As described above, ESCRT machinery might contribute to the release of other types of EVs besides exosomes, because it is involved in remodeling of plasma membrance (Assil et al., 2015). To date, many studies have reported that viral assembly and EVs biogenesis share some similarities, implying that the “hijacking” behavior of viruses in host cells could be in charge of sorting the viral proteins and RNAs into EVs. HIV is a typical example, it can assemble and release from infected cells via ESCRT machinery (Schorey et al., 2015). Influenza viruses bud from the plasma membrane of host cells similar to microvesicles (Lakdawala, 2019), and the similarities of protein profile between influenza virions and microvesicles secreted by uninfected cells have been confirmed by Hutchinson and colleagues, suggesting that influenza viruses can manipulate certain pathway normally used for microvesicles formation (Hutchinson et al., 2014). However, current evidences have suggested that no core ESCRT component involved in IAV particles assembly and budding, implying influenza virus may utilize the ESCRT-independent pathways to facilitate transportation of virus particles to the plasma membrane for release (Bruce et al., 2009; Watanabe and Lamb, 2010; Alenquer and Amorim, 2015). Bruce E. A. et al. (2010) found that Rab 11 involved in the formation of virus particles of IAV by budding from the apical plasma membrane. Morphologically, the virus particles of IAV can take the shape of pleomorphic spheres or vastly elongated filaments. In the study of Bruce et al., Rab11 depletion caused defective budding, low formation of filamentous virions, and virus particles failed to pinch off from the plasma membrane, leading to virus particles apparently stalled in the process of budding. Those results suggested that Rab11 pathway was important in directing vesicular traffic, and influenza virus utilizes the Rab11 pathway for budding. Thereafter, other scholars suggested that Rab 11 was a critical host factor with an essential contribution to transport of vRNPs cargo to the plasma membrane in IAV-infected cells, revealing the potential mechanism of influenza virus genome delivery via a Rab11-dependent vesicular transport pathway (Amorim et al., 2011; Eisfeld et al., 2011). Given Rab family proteins are essential regulators of intracellular vesicle transport between different compartments and vesicle budding, especially RAB11 was the first Rab reported to be involved in exosome secretion (Colombo et al., 2014). Existing studies have shown that the Rab 11 vesicle trafficking is one of the similarities in EVs biogenesis and release of influenza virus particles, implying that influenza virus RNPs may access a Rab11-dependent vesicular transport pathway in recycling endosome (RE) that contributes to the budding of influenza virus particles via EVs secretion.

## Role of EVs in Host Immune Responese for Influenza Virus Infection

During the viral infection, host innate and acquired immune responses can be activated by virus-derived products, accompanied by the recruitment and activation of eukocytes and other cells by cytokines, chemokines, and inflammatory mediators released from infected or resident immune cells, eliciting antiviral action (Schorey et al., 2015). Innate immunity provides the first line of defense and triggers pro-inflammatory responses, while adaptive immunity eliminates the viruses during the later stages of infection (Chen et al., 2018). The recognition and respones to viruses are very important for innate immune system. The viral components such as proteins, lipids, carbohydrates and genetic material, are generally defined as pathogen associated molecular patterns (PAMPs). PAMPs can bind with pattern recognition receptors (PRRs) on the plasma membrane of host cells or dendritic cells (DCs) and MΦ to induce cell-signaling cascades, culminating in the activation of innate immune response against the viruses (Kouwaki et al., 2017). PRRs include Toll-like receptors (TLRs), RIG-I-like receptors, NOD-like receptors, and C-type lectin receptors. Interestingly, viruses have evolved special strategies to evade and/or inhibit host immune responses to promote their virulence and evade immune surveillance. Influenza viruses evolved multiple ways to escape from the host immunity, for instance, some multi-functional proteins such as non-structural protein-1 (NS1) protein and PB1-F2 protein were produced to suppress innate immune signaling pathways (Chen et al., 2018). Moreover, EVs may evoke a “Trojan horse” ploy to help influenza viruses avoid the immune surveillance and favor viral entry into the recipient cells, due to their lower immunogenicity and better biocompatibility. Notably, EVs provide shelter for host and viral proteins and genome by protecting them from DNase, RNase and proteinases in extracellular environment. Some host miRNAs with function of promoting virus replication were found in EVs during influenza virus infection (Keshavarz et al., 2018). For example, miR-17-5p highly expressed in EVs derived from IAV-infected lung epithelial cells and patients' bronchoalveolar lavage fluid (BALF) can result in decreased expression of the antiviral factor Mx1 and significantly enhanced IAV replication (Scheller et al., 2019). Therefore, some scholars speculated that those miRNAs within EVs could also promote influenza virus replication in recipient cells (Keshavarz et al., 2018; Zheng et al., 2020).

In contrast, EVs also are found as an antiviral mediator participating in antiviral immunity. Virus-infected cells can release EVs with functional viral RNAs (e.g., mRNA and miRNA) to DCs and MΦ, and then the viral RNAs are internalized and recognized by PRRs to produce a large quantity of type I interferon and pro-inflammatory cytokines. For example, IAV-RNA can be recognized by TLR7 in plasmacytoid DCs (pDCs), triggering the signal to induce antiviral innate immune responses (Diebold et al., 2004). Recent studies have revealed that infected epithelial cells release EVs that specifically regulate responses of neighboring epithelial cells and immune cells to limit the virus's transmission. Liu et al. (2019) observed that hsa-miR-1975, a Y5 RNA-derived small RNA, was activated in the apoptosis process of influenza virus-infected human lung adenocarcinoma epithelial A549 cells. Subsequently, hsa-miR-1975 was delivered into neighbor cells by exosomes, and fused with other antiviral proteins or nucleotides to produce interferon, and thereby inhibited influenza virus replication when viruses invaded the recipient cells. Besides the infected cells, respiratory epithelial cells not only produce cytokines and chemokines that communicate with immune cells to activate and regulate antiviral responses (Miura, 2019), but also secrete EVs to induce the antiviral responses. Kesimer et al. (2009) firstly observed that human tracheobronchial epithelial (HTBE) cells derived exosome-like vesicles with characteristic exosomal size (30–100 nm) and morphology (cup-shaped), and multivesicular and late endosomal membrane markers Tsg101 and CD63, can neutralized influenza viruses by mean of α-2,6 linked sialic acid on the their surface which can preferentially be bound to influenza viruses, implying an antiviral role for exosomes in mucosal innate defense. Thereafter, Maemura et al. (2018) firstly examined the presence of exosomes in BALF of influenza virus-infected mice, and found the quantity of exosomes enriched miR-483-3p was increased. Those miR-483-3p-containing exosomes might mainly derived form MΦ, but not lung tissues. After exosomal miR-483-3p transfection in lung epithelial cells, the expressions of type I interferon and proinflammatory cytokine were increased by miR-483-3p targeting negative regulators of the RIG-I signaling pathway. Besides BALF, furthermore, high levels of exosomal miR-483-3p was also found in serum of influenza virus-infected mice, and high inflammatory cytokines in vascular endothelial cells (Maemura et al., 2020). Meanwhile, microparticles were found in the BALF of relatively normal subjects who underwent bronchoscopy and bronchoalveolar lavage in the study by Suptawiwat et al. (2017). Those vesicles were originated from bronchial epithelial cells and alveolar epithelial cells, and might exert their anti-influenza activity by trapping influenza virions using their surface sialic acid. The further study have shown that transformed bronchial epithelial BEAS-2B cells enriched both a-2,6- and a-2,3-linked sialic acids, and their microparticles could combat both H1N1 and H5N1, while human lung alveolar epithelial A549 cells only enriched a-2,6-linked sialic acid and their microparticles could only combat H1N1 virus (Jantaratrirat et al., 2018). Additionally, EVs from immune cells are also involved in inflammatory responses to viral infection. Huo et al. demonstrated that mast cells may support the productive replication of influenza virus such as H1N1, H5N1, and H7N2 in their previous studies, however, they observed that exosomes were preferentially secreted from H1N1 or H7N2-infected mouse mastocytoma cell in follow-up study, and speculated that those exosomes were potentially pivotal in innate immunity to fight IAV infection via triggering the robust innate immunity of cells (Huo et al., 2019).

The above findings demonstrate that EVs containing viral components are two sides of the same coin. EVs not only offer convenience to influenza virus replication and immune evasion, and also act as antigen carriers to promoting an innate and acquired immune response to control infection. This enigmatic dual roles of EVs may occur simultaneously and in dynamic balance, that seems to depend on the origin cells, recipient cells and likely many other as well as environmental factors yet undefined factors.

## EVs as an Emerging Vaccine Carriers for Influenza Virus Infection

The occurrence of new influenza viral strains by continually antigenic variation of influenza viruses usually make them resistant to currently used antiviral drugs. Vaccination is the best prophylactic measures for combating influenza virus infection (Okamoto et al., 2018), and recommended for all residents age 6 months and older regardless of the state of their health in United States. During the 2018–2019 influenza season, in United States, the overall adjusted vaccine effectiveness (VE) against all influenza virus infection associated with medically attended ARI was 47%, and VE was 61% in children aged 6 months−17years. VE was estimated to be 74% against illness caused by H1N1, and 26% against H3N2 (Doyle et al., 2019). It is estimated that vaccination could prevent 300–4,000 deaths annually in the United States alone, however, vaccination rates remain low, only 37% of employed adults were vaccinated in 2016 (Mossad, 2018). The currently available influenza vaccines, including inactivated viral particles, M2e-based vaccine, live attenuated influenza vaccine (LAIV) and virus like particle (VLP), are effective against influenza pandemic (Keshavarz et al., 2019). LAIV and VLP vaccines can stimulate both humoral and cellular immune responses, while inactivated vaccines can only induce systemic humoral responses. With the emergence of new influenza viral strains each year, these above vaccines just can provide limited protection. Exosome-based vaccines have been regarded as a new platform as influenza vaccines which have many advantages over traditional vaccines produced in cell culture or eggs (Jungbauer, 2018). For example, avoiding glycosylation directly affect recombinant proteins immunogenicity in avian eggs or endogenous viruses in avian-derived cell lines interfere with the structure of the introduced exogenous virus, causing an allergic reaction after the vaccination. Anticoli et al. (2018) employed an exosome-based vaccine platform to elicite a cytotoxic T lymphocyte (CTL) immunity against influenza virus. Murine muscle cells were transfected with DNA vectors expressing the Nef mutant/Influenza virus A-NP (Nef^mut^/Flu-NP) in this subject, and the murine muscle cell-derived exosomes were purified and injected in mice can lead to a well detectable antigen-specific CD8^+^ T cell response associating with a cytotoxic activity potent enough to kill peptide-loaded and/ or antigen-expressing syngeneic cells, suggesting that this kind of vaccine platform was applicable for further pre-clinical and clinical investigations or applications. Schorey et al. have enumerated the potential advantages to using exosomes as vaccines in their review (Schorey et al., 2015), (1) exosomes are capable of providing a more stable conformational conditions for the proteins. (2) the ability of exosomes to circulate and reach distal organs can improve molecular biodistribution in body fluids. Thirdly, the expression of adhesion molecules on exosomes can provid a more efficient presentation to the antigen-presenting cells. (3) exosomes act one of the body's “natural” mechanisms to transpor antigens between cells, and may play a role in cross-priming. Interestingly, EVs from Gram-negative bacteria, what are now referred to as outer membrane vesicles (OMVs) (Coelho and Casadevall, 2019), have received increased attention as emerging and feasible vaccine carriers (Acevedo et al., 2014; Wang et al., 2018; Yu et al., 2018; Kis et al., 2019). Numerous studies have shown that OMV-derived vaccines could induce protective immunity against influenza viruses (Rappazzo et al., 2016; Lee et al., 2017; Watkins et al., 2017).

## Summary and Outlook

Overall, current evidences suggest EVs play an important role in influenza virus infection. During the process of influenza virus infection, EVs can deliver virus particles that serve as regulators of host defense and mediators of immune evasion, or that serve as antigens of innate immune receptors to stimulate host defense and immunity. Moreover, Exosomes could potentially be used as cell-free vaccines to help people prevent influenza. Many scholars suggested that EVs can not only be a source of diagnostic markers in influenza virus infection, and may also be used as a kind of cell-free therapy, for instance, isolated EVs from swine bone marrow-derived mesenchymal stem cells (MSCs) could inhibit the HA activity of influenza viruses and suppress the replication of influenza viruses (Khatri et al., 2018).

So far, however, some limitation would still affect the EVs studies. For example, nomenclature of EVs have been originated in many literature in accordance with their size, origin and functions, such as microvesicles, microparticles, exosomes, ectosomes, oncosomes, and so on (Colombo et al., 2014). Jurj and colleagues had a detailed introduces of the classification of EVs and their main characteristics (e.g., size, appearance, marker, release process, pathways, etc.) in their recently published review (Jurj et al., 2020). On the basis of the recently updated guidelines of ISEV, “EVs” was defined as the “generic term for particles naturally released from the cell that are delimited by a lipid bilayer and cannot replicate,” but not all EVs researchers agree with the nomenclature of EVs (Théry et al., 2018; Witwer and Théry, 2019). Hence, the exact meaning of EVs cannot be discerned immediately from the names' constituent parts without further explanation. Heterogeneitythe is an inevitable challenge associated with the studies of EVs, which may explain to the confusing and sometimes even conflicting results between laboratories (Suptawiwat et al., 2017; Maemura et al., 2018). Exosome and other EVs usually have various compositions and size will vary depending on their origin, donor cell culture conditions (e.g., glucose levels, fetal bovine serum, antibiotics, mycoplasma and other microbes.) and even physiological or pathological environment of donor cells or tissues including hypoxia, hyperthermia, infections, circadian rhythms, hormones, and stage of cell cycle, etc. (Burger et al., 2017; Németh et al., 2017; Gaurivaud et al., 2018; Ludwig et al., 2019; Pegtel and Gould, 2019; Kalluri and LeBleu, 2020; Zubair et al., 2020). Moreover, the existing isolation and purification technologies (e.g., ultracentrifugation, nanoscale flow cytometry, immunoprecipitation/affinity capture, Exosome Isolation Reagent, etc.) do not meet the special requirements in isolate large quantities of pure and specific EVs from mixtures of different vesicle types in cell culture medium or body fluids (McNamara and Dittmer, 2019). Notably, influenza virus particles and EVs share similarities in density, diameter, etc (McNamara and Dittmer, 2019). Thus, it is sometimes difficult to discriminate the roles of exosomes and other EVs in influenza virus infection and completely explain the specific role of EVs' subtype.

More importantly, some questions such as double-sided actions of EVs in influenza virus infection, and their role in cytokine storm frequently accompanied by ARDS, and even the stability and safety of exosome-based vaccines with foreign antigens on the recipient remain clarified. Therefore, further studies are still needed to investigate EVs composition and function during the pathogenesis process of influenza virus infection.

## Author Contributions

YJ, CX, and WL contributed to the conception and design of the study. YJ wrote the first draft of the manuscript. LY, HG, and JY wrote sections of the manuscript. CX proofread the manuscript. All authors contributed to manuscript revision and approved the submitted version.

## Conflict of Interest

The authors declare that the research was conducted in the absence of any commercial or financial relationships that could be construed as a potential conflict of interest.
